# Unmet need for heart transplantation in Africa

**DOI:** 10.1097/MS9.0000000000002311

**Published:** 2024-06-24

**Authors:** Victor O. Femi-Lawal, Achanga Bill-Smith Anyinkeng, Victory B. Effiom

**Affiliations:** a College of Medicine, University of Ibadan, Ibadan Nigeria; b Faculty of Clinical Sciences, University of Calabar, Calabar, Nigeria; c Department of Research, Association of Future African Cardiothoracic Surgeons, Yaounde, Cameroon; d Faculty of Health Sciences, University of Buea, Buea, Cameroon

**Keywords:** Africa, cardiac surgery, heart failure, heart transplantation, non-communicable disease

## Abstract

Heart transplantation is a critical treatment option for end-stage heart failure patients, offering a lifeline for those with severe cardiac conditions. However, in Africa, the unmet need for heart transplantation is a significant issue that poses challenges to the healthcare system and patient outcomes. Africa faces multiple barriers to heart transplantation, including limited infrastructure, a shortage of skilled healthcare professionals, a lack of funding, and inadequate organ donation systems. These challenges result in a considerable gap between the demand for heart transplants and the available resources to meet this need. As a result, many patients in Africa do not have access to life-saving heart transplantation procedures, leading to high mortality rates among those awaiting transplants. Addressing the unmet need for heart transplantation in Africa requires a multifaceted approach. The authors recommend that Africa as a continent build up a heart transplantation workforce involving a multidisciplinary team that consists of transplant surgeons, transplant physicians, nurses, anesthetists, pharmacists, etc. Heart transplant education and training programs should be well-constructed to ensure the delivery of safe and effective transplantation services. International collaborations have proven to be effective and should be encouraged between African institutions and transplant centers worldwide to facilitate knowledge transfer. Foreign and local organizations should promote public awareness about organ donation to address the myths about heart transplantation and promote heart donation. With these, African countries can improve access to heart transplantation, enhance patient outcomes, save lives in the region, and ultimately reduce the mortality rate in Africa.

## Introduction

Highlights

- In Africa, the practice of heart transplantation is significantly low, compared to the rest of the world.
- Only a few countries can support heart transplantation due to a paucity of skilled professionals, a lack of funding, and inadequate organ donation systems.
- Suggestions for improving transplantation include increasing investment in infrastructure and personnel development, establishment of organ procurement and transplant systems, and improving public awareness.

Human-to-human heart transplantation, which is the replacement of a failing heart with one from a suitable donor, was first carried out by Dr Christian Barnard at the Groote Schuur Hospital in Cape Town, South Africa^1^. This was a victory for medicine globally and also suggested the potential of Africa as a center for medical innovation. Today, over 6000 heart transplants are conducted annually, and recipients have a one-year survival rate of nearly 90%^2,3^. As specified by guidelines from the American College of Cardiology, American Heart Association, Heart Failure Society of America, and European Society of Cardiology, the procedure is typically conducted for patients with diseases such as cardiomyopathies, coronary heart failure, and congenital heart diseases that are refractory to conventional therapy^4^.

Significant progress has also been made in the evolution of heart transplantation, with advances in immunosuppression, noninvasive imaging techniques like transthoracic echocardiography, and screening to prevent acute and chronic rejection of transplants^5^. In this interim, Africa appears to have been left behind in the development of the field of heart transplantation. However, to our knowledge, studies have yet to document the extent of this gap. This review highlights the existing gaps and unmet need for heart transplantation in Africa, exploring the challenges and possible solutions.

## Current state of heart transplantation in Africa

The practice of heart transplantation in Africa, with a population of over 1.2 billion and 54 countries, vastly underwhelms the need^6^. According to reports, cardiovascular diseases are responsible for 38% of all non-communicable disease deaths in Africa, contributing to a double burden of infectious and non-communicable diseases^7^. These are conservative estimates, however, as most data is from urban centers in a few countries. In some cases, this data is non-existent^8^. According to the Global Observatory of Donation and Transplantation 2017 database, Africa recorded only 14 heart transplants out of 7881 globally, representing 0.2% of the global total in 2016^9^. Similarly, in 2022, the International Report on Organ Donation and Transplantation Activities recorded heart transplantations in only one country in Africa, Tunisia^10^.

The practice of heart transplantation in Africa has historically been limited to a few centers in select countries. Currently, countries such as South Africa, Tunisia, Namibia, and Uganda represent the only territories with heart transplant procedures. The Groote Schuur Hospital in Cape Town, the cradle of heart transplantation, has been one of the most consistent centers for heart transplantation in Africa. Heart transplantation has continued without interruption at this center since the first one in 1967, with heterotopic and orthotopic methods primarily utilized. The team at this center has also engaged in research on cardiac transplantation, advancing concepts such as brain death, organ and tissue donation, and ethical issues^9^. Records at this center show that early graft failure is a key cause of morbidity and mortality^1^. The country has the most advanced transplant program in Africa^11^. Tunisia is another center where heart transplants have been conducted–10 were conducted between 1993 and 2004—although numbers have declined with time, with economic costs and logistics cited as a cause of the low numbers^12,13^. In 2023, the Ministry of Health in Tunisia announced that the Rabta University Hospital in Tunis performed its 21^st^ heart transplant, a positive development in cardiothoracic surgery in the country^14^.

Countries such as Namibia and Uganda, with one center each, have also been recorded to have performed heart transplants at some point, although scientific reports of the procedure have lately diminished^15^. Egypt has a thriving organ transplantation practice, though reports of heart transplantation are sparse in the scientific record. Nigeria, another country with established organ donation and transplantation centers, has yet to publish a record of heart transplantation. Overall, the records suggest that while heart transplantation is offered in a few countries in Africa, their activity is much lower than that of countries with more economic capacity^16^. In 2020, the Global Report on Organ Transplantation and Donation showed that as of that year, no heart transplant had been carried out in any African country, and there was no heart transplant center in any of the African countries to meet the needs of the population^17^.

This is really a serious problem because Africa is home to greater than 1 billion people, and is a major contributor to the global burden of cardiovascular diseases (CVDs) and other heart-related problems^18^. In fact, in 2013, it was estimated that 1 million deaths were attributable to CVD in sub-Saharan Africa alone, which made up about 5.5% of all global CVD-related deaths and 11.3% of all deaths in Africa^19^. Furthermore, CVD-related deaths contributed to about 38% of all non-communicable disease-related deaths in the African region, reflecting the growing threat of both non-communicable diseases and CVD^19^. As opposed to several high-income countries (HIC), which have recorded reductions in cardiovascular deaths^20^, Africa as a continent has registered about a 50% increase in the CVD burden within the last three decades^21,22^. Some of these cardiovascular conditions would have been averted just with heart transplantation, hence the growing need for heart transplantation in this region.

## Barriers to heart transplantation in Africa

Heart transplantation is considered one of the most effective treatments for end-stage heart failure, giving patients another chance for survival and an improved quality of life. However, this practice faces impediments in Africa due to various barriers, including inadequate infrastructure, a lack of personnel for such procedures, a shortage of organ donors, negative beliefs regarding organ donation, and economic constraints.

The infrastructure required to support heart transplantation is often unavailable in many African countries. This includes specialized cardiac centers with dedicated intensive care units, sophisticated diagnostic materials, surgical equipment, tissue typing, cross-matching, and some viral studies^23^, which are essential for pre- and post-transplantation care. This delays the process and affects timely action on tissue rejections and infection. A study conducted by Edwin and colleagues in 2016 on the perceived barriers to and facilitators of physical activity in recipients of solid organ transplantation showed that one of the reasons for poor outcomes in patients with heart transplant surgery was the unavailability of equipment^24^

The inadequate availability of transplant surgeons and support staff (pathologists, internal medicine physicians, and surgical nurses) remains a significant setback, limiting the number of transplantations even in centers with adequate facilities. This impedes the growth of transplantation in Africa, given the stark contrast of only 2.3 healthcare workers for every 1000 population in Africa, as opposed to 24.8 healthcare workers for every 1000 population in America^23,25^. In 2015, Ziz and colleagues, in a report on the update of organ transplantation in Tunisia, a study that covered both heart and kidney transplantation, showed that the unavailability and lack of motivation of medical personnel were one of the limitations to carrying out transplant surgeries in the country^26^

Insufficient legal and regulatory frameworks, coupled with cultural beliefs and religious views regarding heart transplantation, pose significant obstacles in Africa. Rigid legal frameworks also halt the progression of this practice^25,27^, and the absence of an established organ procurement and allocation system further complicates the process of matching organ donors with recipients and avoiding organ trafficking. Jose-Maria *et al*.^28^, in their 2023 publication, revealed that cultural and religious beliefs were a major challenge to heart transplantation due to the fact that people believed that heart donation was against their faith and their cultural norms.

The financial costs associated with the procedure, including the preoperative workup, surgery, postoperative care, and immunosuppressive therapy, inflict a great burden on the patient and healthcare facilities. The unavailability of funding also becomes a significant obstacle in implementing this program in most African countries^23^. In fact, in 2021, it was reported in Uganda that one of the major barriers to performing a heart transplant was the financial challenge it poses on the part of the patient and the medical facility due to it being an expensive procedure^29^

Heart transplantation holds a promising future for end-stage heart pathologies in Africa. However, there are still major setbacks in its implementation. Resolving the above-mentioned barriers will be crucial for improving access to and increasing the success rate of transplantation.

## Changing the landscape: recommendations for improving the penetration of heart transplantation in Africa

Improving the penetration of heart transplantation in Africa will require comprehensive and close collaboration with national and international organizations, non-governmental organizations, and healthcare staff. The following recommendations are vital in enhancing the penetration, accessibility, and success of heart transplantation in Africa.

Public sensitization and education are crucial parts of establishing heart transplant programs, with Africa faced with diverse and conflicting interests (religion, cultural belief, and social norms) regarding the notion of organ transplantation, especially the heart. Therefore, public education campaigns tailored to factors such as age, profession, education level, cultural sensitivity, and religious affiliation, along with community outreach using key community members and partnership programs with churches and community leaders, can help alleviate the dilemmas associated with this practice. This, in turn, can increase the pool of potential donors and improve the acceptability of heart transplantation to recipients.

Training and capacity-building to improve local capacity in African transplant medicine are vital. This will involve expanding existing programs and creating new training programs for medical professionals in cardiac surgery, transplant immunology, and post-transplant care. This will require collaboration with other well-established international transplant centers and societies for mentorship and technical assistance while facilitating knowledge transfer, exchange of expertise, and collaborative research.

Infrastructure development is critical to actualizing the penetration and implementation of heart surgery in Africa due to the huge deficit in infrastructure to support cardiac procedures. Therefore, the building of specialized cardiac centers equipped with standard facilities and equipment for cardiac surgery, intensive care units, and post-transplant care is essential to supporting the growth of heart transplantation programs in Africa.

Health insurance and financing reforms are still underdeveloped in Africa. With the low-income status of the inhabitants, out-of-pocket payments are a huge burden. Thus, health insurance and financial reforms are needed to expand to include the coverage of advanced medical procedures such as heart transplantation. This could be done by developing specific insurance programs or subsidies to promote access to transplant services for eligible patients. The support of NGOs and philanthropists will also be instrumental in the interim.

Organ procurement and allocation centers and systems are key to establishing an efficient transplant system. This will involve developing regulatory frameworks, national registries accessible to all transplant centers, and regulatory boards collaborating with transplant networks to ensure equity and ethical distribution of donor organs. This will equally involve advocating for policy changes and legislation that support and regulate organ donation, transplantation, and post-transplant care.

## Conclusion

Heart transplantation in Africa is far below the demand for the procedure, only practiced in a handful of countries. Challenges to the practice range from weak infrastructure to inadequate surgical capacity to a lack of financial capacity on the part of the patients. We recommend that Africa build up its heart transplantation workforce by involving a multidisciplinary team that consists of heart transplant surgeons, heart transplant physicians, nurses, pharmacists, anesthetists, social workers, dietitians, counselors and clinical coordinators. Heart transplant education and training programs should be well-constructed to ensure the safe and effective delivery of heart transplantation services. International collaborations should be encouraged between African institutions and health transplant centers worldwide to facilitate knowledge transfer. Non-governmental organizations and government agencies in various African countries should promote public awareness about heart donation to address the myths about heart transplantation and promote heart donation. These would help to improve heart transplantation in the African continent.

## Ethics statement

Not applicable.

## Consent

Patient data were not collected, so this does not apply to this study.

## Source of funding

No funding was received for this work.

## Author contribution

V.O.F.-L., A.B.-S., and V.B.E. conceptualized this work. V.O.F.-L. and A.B.-S. drafted the manuscript. V.O.F.-L., A.B.-S., and V.E. contributed to the editing and critical revision of this work. All authors read and approved the final draft.

## Conflicts of interest disclosure

The authors declare that they have no known competing financial interests or personal relationships that could have appeared to influence the work reported in this paper.

## Research registration unique identifying number (UIN)

Not applicable.

## Guarantor

Victor O. Femi-Lawal.

## References

[1] Hassoulas J. Transplantation of the heart: an overview of 40 years’ clinical and research experience at Groote Schuur Hospital and the University of Cape Town: Part I. Surgical experience and clinical studies. S Afr Med J 2012;102(6 Pt 2):350–352. 22668902 10.7196/samj.5020

[2] Khush KK Hsich E Potena L. The International Thoracic Organ Transplant Registry of the International Society for Heart and Lung Transplantation: Thirty-eighth adult heart transplantation report — 2021; Focus on recipient characteristics. *J Heart Lung Transplant* 2021;40:1035–1049. 34419370 10.1016/j.healun.2021.07.015 PMC10282986

[3] Kittleson MM Kobashigawa JA. Cardiac transplantation. Curr Outcomes Contemp Controversies JACC Heart Fail 2017;5:857–868. 10.1016/j.jchf.2017.08.021 29191293

[4] DiNella JV Bowman J. Heart transplantation. Crit Care Nursing Clin North Am 2011;23:471–479. 10.1016/j.ccell.2011.08.005 22054822

[5] Weber BN Kobashigawa JA Givertz MM. Evolving areas in heart transplantation. JACC Heart Fail 2017;5:869–878. 29191294 10.1016/j.jchf.2017.10.009

[6] Obonyo NG Etyang AO. Cardiovascular health priorities in Sub-Saharan Africa. SN Compr Clin Med 2023;5:262.

[7] Keates AK Mocumbi AO Ntsekhe M. Cardiovascular disease in Africa: epidemiological profile and challenges. Nat Rev Cardiol 2017;14:273–293. 28230175 10.1038/nrcardio.2017.19

[8] Kwan GF Mayosi BM Mocumbi AO. Endemic cardiovascular diseases of the poorest billion. Circulation 2016;133:2561–2575. 27297348 10.1161/CIRCULATIONAHA.116.008731

[9] Ulasi I Ijoma C Ifebunandu N. Organ donation and transplantation in Sub-Saharan Africa: opportunities and challenges. Organ Donation and Transplantation. IntechOpen; 2020.

[10] Global Observatory on Donation and Transplantation. International Report on Organ Donation and Transplantation Activities 2022. Published online 2023. Accessed February 25, 2024. https://www.transplant-observatory.org/wp-content/uploads/2023/11/2022-data-global-report_VF_2.pdf.

[11] Moosa MR. The state of kidney transplantation in South Africa. South Afric Med J 2019;109:235–240. 10.7196/SAMJ.2019.v109i4.13548 31084688

[12] El Matri A Ben Abdallah T. Organ transplantation in Tunisia. Exp Clin Transplant 2015;13(suppl 1):33–36. 25894125

[13] Drissa M Drissa H Helali S. Epidemiology and management of heart failure with reduced ejection fraction in a Tunisian university hospital. Cardiovasc J Afr 2023;34:68–72. 37132406 10.5830/CVJA-2018-070 PMC10512042

[14] Tunisia: Successful Heart Transplant Performed At Rabta Hospital in Tunis. *Tunis Afrique Presse* Accessed February 25, 2024. https://allafrica.com/stories/202301090249.html

[15] WHO. Status of Human Organ and Tissue Donation and Transplantation in the WHO African Region: Report of the Secretariat. Published online 2020. Accessed February 25, 2024. https://www.afro.who.int/sites/default/files/2021-02/AFR-RC70-12%20Status%20of%20human%20organ%20and%20tissue%20donation%20and%20transplantation.pdf

[16] Delmonico FL Domínguez-Gil B Matesanz R. A call for government accountability to achieve national self-sufficiency in organ donation and transplantation. Lancet 2011;378:1414–1418. 22000137 10.1016/S0140-6736(11)61486-4

[17] Global report on organ donation and transplantation 2020. https://www.transplant-observatory.org/wp-content/uploads/2022/07/2020-Global-report-para-web.pdf

[18] Moran A. The epidemiology of cardiovascular diseases in sub-Saharan Africa: the Global Burden of Diseases, Injuries and Risk Factors 2010. Study Prog Cardiovasc Dis 2013;56:234–239. 24267430 10.1016/j.pcad.2013.09.019 PMC4031901

[19] Mensah GA. Mortality from cardiovascular diseases in sub-Saharan Africa, 1990-2013: a systematic analysis of data from the Global Burden of Disease Study 2013. Cardiovasc J Afr 2015;26:S6–S10. 25962950 10.5830/CVJA-2015-036 PMC4557490

[20] Sacco RL Roth GA Reddy KS. The heart of 25 by 25: achieving the goal of reducing global and regional premature deaths from cardiovascular diseases and stroke: a modeling study from the American heart association and world heart federation. Circulation 2016;133:e674–e690. 27162236 10.1161/CIR.0000000000000395

[21] Institute for Health Metrics and Evaluation [IHME]. GBD Compare Data Visualization. (2020). Accessed April 21, 2022. http://vizhub.healthdata.org/gbdcompare

[22] Gouda HN Charlson F Sorsdahl K. Burden of non-communicable diseases in sub-Saharan Africa, 1990–2017: results from the Global Burden of Disease Study 2017. Lancet Glob Health 2019;7:e1375–e1387. 31537368 10.1016/S2214-109X(19)30374-2

[23] Awuah WA Ng JC Bulut HI. The unmet need of organ transplantation in Africa. Int J Surg 2023;109:519–520. 36927835 10.1097/JS9.0000000000000025 PMC10389210

[24] van Adrichem EJ van de Zande SC Dekker R. Perceived barriers to and facilitators of physical activity in recipients of solid organ transplantation, a qualitative study. PLoS One 2016;11:e0162725. 27622291 10.1371/journal.pone.0162725 PMC5021267

[25] Loua A Feroleto M Sougou A. A review of policies and programmes for human organ and tissue donations and transplantations, WHO African Region. Bull World Health Organ 2020;98:420–425. 32514216 10.2471/BLT.19.236992 PMC7265924

[26] El Matri A Ben Abdallah T. Organ transplantation in Tunisia. Exp Clin Transplant 2015;13(suppl 1):33–36. 25894125

[27] Pullen LC. Transplantation in Africa. Am J Transplant 2017;17:1431–1432. 28544283 10.1111/ajt.14331

[28] Dominguez-Roldan Jose-Maria Haq Ikran ul Ahmed N. Cultural and religious aspects of heart transplantation. Springer 2022:271-293.

[29] Heart Transplantation - Uganda Heart Institute. Published September 27, 2021. Accessed April 24, 2024. http://www.uhi.go.ug/heart-transplantation/

